# Biomarker expression level changes within rectal gut-associated lymphoid tissues in spinal cord-injured rats

**DOI:** 10.1093/immhor/vlaf002

**Published:** 2025-03-06

**Authors:** Yun Zhou, Charles H Hubscher

**Affiliations:** Department of Anatomical Sciences and Neurobiology, University of Louisville School of Medicine, Louisville, KY, United States; Kentucky Spinal Cord Injury Research Center, Louisville, KY, United States; Department of Anatomical Sciences and Neurobiology, University of Louisville School of Medicine, Louisville, KY, United States; Kentucky Spinal Cord Injury Research Center, Louisville, KY, United States

**Keywords:** biomarker, gut-associated lymphoid tissue (GALT), gut microbiome dysbiosis (gut dysbiosis), inflammation, neurogenic bowel dysfunction (NBD)

## Abstract

Neurogenic bowel dysfunction (NBD) is common after spinal cord injury (SCI). Gut-associated lymphoid tissue (GALT), an organized structure within the mucosal immune system, is important for the maintenance of gut homeostasis and body health and serves as the first line barrier/defense against diet antigens, commensal microbiota, pathogens, and toxins in mucosal areas. The current study examined gene expression levels along six segments of anorectal tissue using real-time polymerase chain reaction (RT-PCR) in uninjured rats (28-day sham surgical controls) and at both 28- and 42-days post-T9 contusion injury. Consistent with our previous report of functional regional differences in the ano-rectum, we demonstrate the existence of GALTs located primarily within the segment at 3-4.5 cm from the rectal dentate line (termed rectal GALTs—rGALTs) in shams with upregulated gene expression levels of multiple biomarkers, including B cell and T cell-related genes, major histocompatibility complex (MHC) class II molecules, and germinal center (GC)-related genes, which was further confirmed by histologic examination. In the same rectal tissue segment following T9 SCI, inflammation-related genes were upregulated at 28 days post-injury (DPI) indicating that microbial infection and inflammation of rGALTs modified structure and function of rGALTs, while at 42 DPI rGALTs exhibited resolution of inflammation and impaired structure/function for extrafollicular B cell responses. Taken together, our data suggest that rGALTs exists in rat rectum for homeostasis of gut microbiota/barrier. SCI induces microbial infection and inflammation in rectal tissues containing rGALTs, which could contribute to development of SCI-related gut microbiome dysbiosis, NBD, and systemic diseases.

## Introduction

Neurogenic bowel dysfunction (NBD) is a common source of morbidity and re-hospitalization after traumatic SCI^1^ and ranks high in terms of having a negative impact on daily quality of life.^2–6^ NBD affects the entire gastrointestinal (GI) tract and causes both functional and psychological issues in individuals with spinal cord injury (SCI)^2^^,^^7^^,^^8^ as well as other CNS diseases/conditions including multiple sclerosis (MS)^9^ and stroke.^10–12^ SCI-related NBD includes constipation, fecal incontinence/impaction, difficulty with evacuation, abdominal distention, decreased anorectal sensation and autonomic dysreflexia.^7^^,^^8^^,^^13^ Although research has been limited, several recent studies have focused on identifying cellular and molecular mechanisms underlying NBD after SCI.^14–16^ Growing evidence suggests that due to central nervous system (CNS) injury-induced immunodepression (CIDS),^17^ CNS trauma commonly induces microbial infection and inflammation,^18^^,^^19^ which could be associated with diseases such as neurogenic bladder^20^^,^^21^ and neurogenic bowel dysfunction (NBD).^22^^,^^23^

Gut-associated lymphoid tissue (GALT), belonging to secondary lymphoid organs (SLOs), is an organized immune structure/organ within the mucosal immune system.^24–27^ GALTs are the primary sites of generating IgA^+^ antibody secreting plasma cells (IgA^+^ ASCs) and IgA^+^ memory B cells^28^ for mucosal secretory immunoglobulin A (sIgA). GALTs are indispensable for the maintenance of gut homeostasis and body health as they serve as the first line barrier/defense against diet antigens, commensal microbiota, pathogens and toxins in mucosal areas.^25^^,^^29–38^ The GALTs, as macroscopically visible lymphoid aggregates, include Peyer's patches (PP),^25^^,^^39^ cecal patches,^40^ and colonic lymphoid patches (CLPs).^41^^,^^42^ The GALTs also include solitary isolated lymphoid tissue (SILT),^43^^,^^44^ which ranges in size from small cryptopatches to mature isolated lymphoid follicles (ILF) and can only be detected microscopically.^45–48^ CLPs and SILTs are histologically distributed irregularly along the entire colon in adult mice^40–42^^,^^49^^,^^50^ and rats.^40^^,^^51–53^ In humans, colonic GALTs contain submucosal ILF (SM-ILF) and lamina propria-embedded mucosal ILF (M-ILF) with small or absent germinal centers (GCs),^47^^,^^48^^,^^54^^,^^55^ which provide evidence that, in addition to T cell-independent (TI) IgA generation (TI IgA^+^) in non-lymphoid organs (NLO) in gut,^56–59^ GALTs produce both T cell-dependent (TD) IgA generation (TD IgA^+)^ and TI IgA^+^ depending on cognate T cell help.^37^^,^^43^^,^^44^^,^^58^^,^^60^ Meanwhile, GALTs are structurally connected with mesenteric lymph nodes (MLN) and whole-body system via lymphatic vessels and afferent lymphatics^61^ for migration/homing of lymphoid cells to the lamina propria of the intestine.^56^^,^^62^^,^^63^ Thus, B cells/plasma cells from GALTs play a role for shaping the B cell repertoire,^62^^,^^63^ contribute to bone marrow plasma cell pool^63–65^ and could reside/function in NLO^66^ including CNS.^67–69^ Indeed, SCI induced gut microbiome dysbiosis^22^^,^^23^^,^^70–72^ and disorders of hindgut function,^7^ which could be mediated by dysfunction of the immune structure/organ in the hindgut region as the colorectum contains the highest commensal microbiota at about 10^9^ to 10^12^ microbial load per ml compared to other parts of the intestine.^49^ Although there are some reports in humans^55^^,^^73^^,^^74^ and mice,^42^ existence of rectal GALTs remains to be further elucidated and may have clinical implications for studying rectal diseases including colorectal cancer.^75^^,^^76^

The pre-clinical SCI rat model reproduced symptoms of human SCI subjects and develop gut microbiome dysbiosis/inflammation,^13^^,^^72^^,^^77^ and regional differences in the rat rectum have been demonstrated by using functional anorectal manometry.^13^ In the current study through examining changes of expression levels of immune biomarkers along the colorectal region in rat by real-time polymerase chain reaction (RT-PCR), we have identified the existence of rectal gut-associated lymphoid tissues (termed rectal GALTs—rGALTs) in the local immune system in rat rectum. Through analyzing changes of immune biomarkers within rGALTs at 2 time points post-SCI, we provide evidence that SCI induced impairment of structure and function of rGALTs, which likely relates to ENS and developing SCI-related gut dysbiosis, NBD, and systemic diseases.^7^^,^^47^^,^^78^ The current study illustrates the clinical problem through the study of multiple molecular biomarkers in rat SCI model, and both translational and precision medicine.^79^

## Materials and methods

### Animals 

All animal procedures were performed according to National Institutes of Health guidelines and received approval from the Institutional Animal Use and Care Committee at the University of Louisville, School of Medicine. A total of 30 adult male Wistar rats (Harlan; Sprague Dawley, Inc., Indianapolis, Indiana, USA), weighing initially ∼250 *g*, were used for the current study, as a majority of the SCI population is male. All rats were housed with a 12-hour light-dark cycle with ad libitum food (Laboratory Rodent Diet) and water.

### Spinal cord injuries

Either a laminectomy plus contusion or laminectomy alone (shams, no spinal cord contusion) were performed at the T9 spinal level after removal of the T8 vertebra under ketamine/xylazine anesthesia (80 mg/kg [Fort Dodge Laboratories, Fort Dodge, Iowa, USA]; 10 mg/kg [Akorn Inc, Lake Forest, Illinois, USA], respectively) according to established protocols.^80^ Contusions were made with the Infinite Horizon impactor device (Precision Systems and Instrumentation, Fairfax Station, Virginia. USA) using a force of 225 kilodyne (no dwell time).^81^

### Bowel tissue RNA preparation and RT-PCR

**Figure 1.**
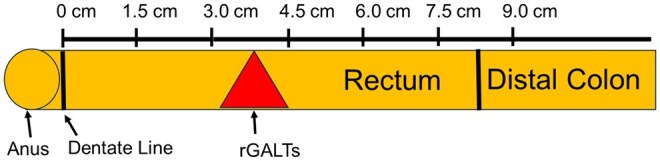
Anatomical diagram of the rat rectum for tissue collection. Rat rectal tissues 9 cm from the rectal dentate line were collected for the current study. The locations of the anus, the dentate line and the GALTs are labeled in this diagram.

Tissues from 18 rats (6 shams, 6 rats at 28-day post-SCI injury group [DPI], 6 rats at 42 DPI) were used for RNA preparation. Post-injury times represent sub-acute and chronic timepoints. Rats were overdosed with 50% urethane (1.2 g/kg ip, Sigma, Missouri, USA) and immediately perfused transcardially with 300 ml exsanguination solution (154 mM sodium chloride, 14.2 mM NaH_2_PO_4,_ 100 mM Na_2_HPO_4_) plus 1 ml Heparin (DV Medical Supply). Anorectal segments including the rectum and part of the distal colon were collected for tissue RNA preparation.^13^ After removing the anal parts, serial anorectal segments from dentate line along the entire anorectum (1.5 cm each, total of 6 segments; Fig. 1) were used to examine expression levels of various targeted genes. RNA was prepared with trizol and purified by filtering through columns (Fisher Scientific). The concentration of RNA was determined by using a NanoDrop spectrophotometer. A high-capacity cDNA reverse transcription kit (Fisher Scientific) was used for RNA transcription to cDNA. RT-PCR reaction mixture (Fisher Scientific) was performed on a QuantStudio™ 3 RT-PCR System (Applied Biosystems Inc, Beverly, Massachusetts, USA). The PCR cycle was as follows: stage1, 50 °C, 2 minutes, 95 °C 10 minutes; stage 2, 95 °C 15 seconds, 60 °C 1 minute, 40 cycles; melt curve, 95 °C 15 seconds, 60 °C 1 minute, 95 °C 15 seconds. The relative quantities of target transcripts were calculated after normalization of the data against a housekeeping gene Actb. Melting curve analyses were performed after PCR amplification to confirm the specificity of the primers. Fold change in gene expression was calculated using the formula 2^-ΔΔCt^. The data are representative of 3 biological replicates. Primers examined are provided in Table 1.

**Table 1.** List of study primers.

Gene name	Forward primer (FW)	Reverse primer (RE)
**Actb**	**5′-CCTCTATGCCAACACAGTGC**	**5′-CACAGAGTACTTGCGCTCAG**
**Ighm**	**5′-AGGATGGGAAGCCTGTGAAA**	**5′-TCAGTGATGGTCAGTGTGCT**
**Ighd**	**5′-CACATTCACCCACCACGAAA**	**5′-GGGTTGTGCAGTGACAAAGT**
**Igha**	**5′-ATACACCATGTGCAGCCAGT**	**5′-CGGCAGACTAAGGAGGGTTT**
**Cd19**	**5′-TGGTGGAGGTAGAAGAGGGA**	**5′-CTCCAGGAAGGGTGTTGACT**
**Slamf7**	**5′-GTCACACTAGTGCTCGTCCT**	**5′-TCTCCAAGTGAGGGCAAGAG**
**Cd38**	**5′-CTCTTGCCCACATTGGAGTG**	**5′-GAGCATCACTTGGACCACAC**
**Cd27**	**5′-GCCAGGGACATTCCTTGTGA**	**5′-GGGTGTGGTAGTCTGGAGAG**
**Cd4**	**5′-AAGGATTCAGGACAGTGGCA**	**5′-TGATGGATGTGCTCGCAAAG**
**Cd40lg**	**5′-AATGGGAGACAACTGACGGT**	**5′-TTCTCTCTGACCCACTGCTG**
**Ptprc**	**5′-CCCGGGATGAGACAGTTGAT**	**5′-TCTGAAAGTCCGAGTGCCTT**
**Ptprc-RA**	**5′-CACATATCTTCCAGGTGCCAAC**	**5′-CGCACAGTAACGTTCCCAAA**
**Ptprc-RB**	**5′-ACCGACGATGGTTTTTCATCCAC**	**5′-TGAGGTTGGCACCTGGTGGGTC**
**Ptprc-RO**	**5′-ACAACCGACGATGGTGCCAAC**	**5′-CGCACAGTAACGTTCCCAAA**
**Sell**	**5′-GGTCATCCCTAGAGCCAGTC**	**5′-GAAGCTGAAGTTGGCCAGAG**
**RT1-Da**	**5′-CGTGTCAGAGACGGTGTTTC**	**5′-CCAGTGATCCACCTCACAGT**
**RT1-Db1**	**5′-TGGTGATGCTGGAGATGGTT**	**5′-GACTGTGCTTTCCACTCGAC**
**RT1-Ba**	**5′-CCAGCTACCAACAAGGTTCC**	**5′-TGGCTTGCTGTTTCTCAACC**
**RT1-Ha**	**5′-TGACGGTGATGAGCAGTTCT**	**5′-GGCTGTCGATGTAGGGAAGA**
**RT1-DMa**	**5′-CTGCGACATGTTGATGCAGA**	**5′-TGAAACAGACCAGCGTGTTG**
**Ccr6**	**5′-AAAGCCAGGTCCATGACTGA**	**5′-CCAAGTGTCAGTGGCATGAG**
**Cxcl13**	**5′-CACGGTATTCTGGAGACCCA**	**5′-CCATTCCCAGGGCGTATAAC**
**Cxcr5**	**5′-GCCTACAGCCTCATCTTCCT**	**5′-ACAGGAAGGTCTCCGTTGAG**
**Ccl19**	**5′-GCTGGTTCTCTGGACCTTCT**	**5′-ACGATGTTACCAGGGATGGG**
**Ccr7**	**5′-GTGCTTCTGCCAAGATGAGG**	**5′-GGCCTTAAAGTTCCGCACAT**
**Cxcr4**	**5′-CGGTCATCCTTATCCTGGCT**	**5′-GACGCTCTCGAACTCACATC**
**Gpr183**	**5′-ACTTCAACTGCTGCATGGAC**	**5′-TTCCCGTGAGTTCTCCTCAG**
**Mki67**	**5′-AATGCCCAGCAAATCTCCAC**	**5′-TTGCAGGCTCTTCTCTCACA**
**Fas**	**5′-AAAGGTACCGGAAGAGGCAA**	**5′-TTCGGCAGTTCTCCAGATGT**
**Bcl2l11**	**5′-AAGGCAGTCTCAGGAGGAAC**	**5′-TCTTCCGCCTCTCGGTAATC**
**Bax**	**5′-ACTAAAGTGCCCGAGCTGAT**	**5′-AAGATGGTCACTGTCTGCCA**
**Il6**	**5′-TCTCTCCGCAAGAGACTTCC**	**5′-CTGGTCTGTTGTGGGTGGTA**
**Il10**	**5′-GGCTGCCTTCAGTCAAGTG**	**5′-CTTGGCAACCCAAGTAACCC**
**Tlr2**	**5′-TGCAGGGACCTTTGCTATGA**	**5′-TGAAGGGTGGGTCAGAGTTC**
**Il1b**	**5′-ACTCATTGTGGCTGTGGAGA**	**5′-TAGCAGGTCGTCATCATCCC**
**Vcam1**	**5′-GACCTGTCAGCGAAGGAAAC**	**5′-CATTAGGGACCGTGCAGTTG**
**Icam1**	**5′-ACGGAGCCAATTTCTCATGC**	**5′-TCAGGACCCTAGTCGGAAGA**
**Selplg**	**5′-CAATGGTGGCCGTGTTAGAG**	**5′-CCGCTGTGTGACAGATTCAG**
**Ccl21**	**5′-AGCTTGGTCCTGGTTCTCTG**	**5′-CTTCCTGTAGCCTCGGACAA**
**Ccl20**	**5′-CCGACGAAGCTTGTGACATT**	**5′-TTAGGCTGAGGAGGTGCAAA**

### Bowel tissue histology study

Twelve male rats (3 at 28 DPI, 3 at 42 DPI, 6 age-matched intact controls) were anesthetized and perfused transcardially with exsanguination solution and 4% paraformaldehyde in 0.1 M phosphate buffer. Bowel tissue was further fixed in 4% PFA overnight and stored in a 30% sucrose-PBS solution. Per the RT-PCR outcome data, 1.5–3 cm, 3–4.5 cm and 4.5–6 cm segments from dentate line (Fig. 1) were embedded in tissue freezing medium (Fisher Scientific) and transversely sectioned on a cryostat at 30 µm thickness. For hematoxylin and eosin *(*H&E) staining, 12 slides (one from each of the 12 rats) for each segment were rinsed in water to remove tissue embedding compound and stained with hematoxylin for 1 minute. After tap water wash for 10 minutes, slides were then stained with eosin for 1 minute and dehydrated in 70%, 80%, and 95% alcohol for 10 minutes each. Slides were mounted with Permount medium (Fisher Scientific) and covered with cover glasses. Tissues were imaged using a Nikon Eclipse Ti microscope. For immunofluorescence and confocal microscopy, nuclear staining was accomplished with Hoechst 33342 (Life Technologies) at 1:2,000 for 1 hour (a slide from each of the same 12 rats used for H&E stain). Slides were mounted with anti-fade mounting medium (Vector Laboratories, Newark, California, USA) and imaged using a Nikon C2+ confocal microscope.

### Statistical analysis

GraphPad Prism 5.04 (GraphPad Software Inc., San Diego, California, USA) was used for statistical analyses. Results are expressed as means ± SEM. Gene expression for segmental distribution was normalized to the same gene expression at the 0–1.5 cm segment and was analyzed by paired *t* tests (marked as *). Expression level changes of immune biomarkers at the 3–4.5 cm segment between sham and injury groups (28 DPI, 42 DPI) were made by using the corresponding gene’s normalization value at the 3–4.5 cm segment according to the same gene value at the 0–1.5 cm segment; and statistical comparisons were analyzed by 1-way analysis of variance (ANOVA) with Tukey’s post-hoc test correction (marked as *) or unpaired *t* tests (marked as #) where appropriate. Differences were considered statistically significant when *P* < 0.05.

## Results

### Gene expression levels of B cell/plasma cell-related genes

**Figure 2.**
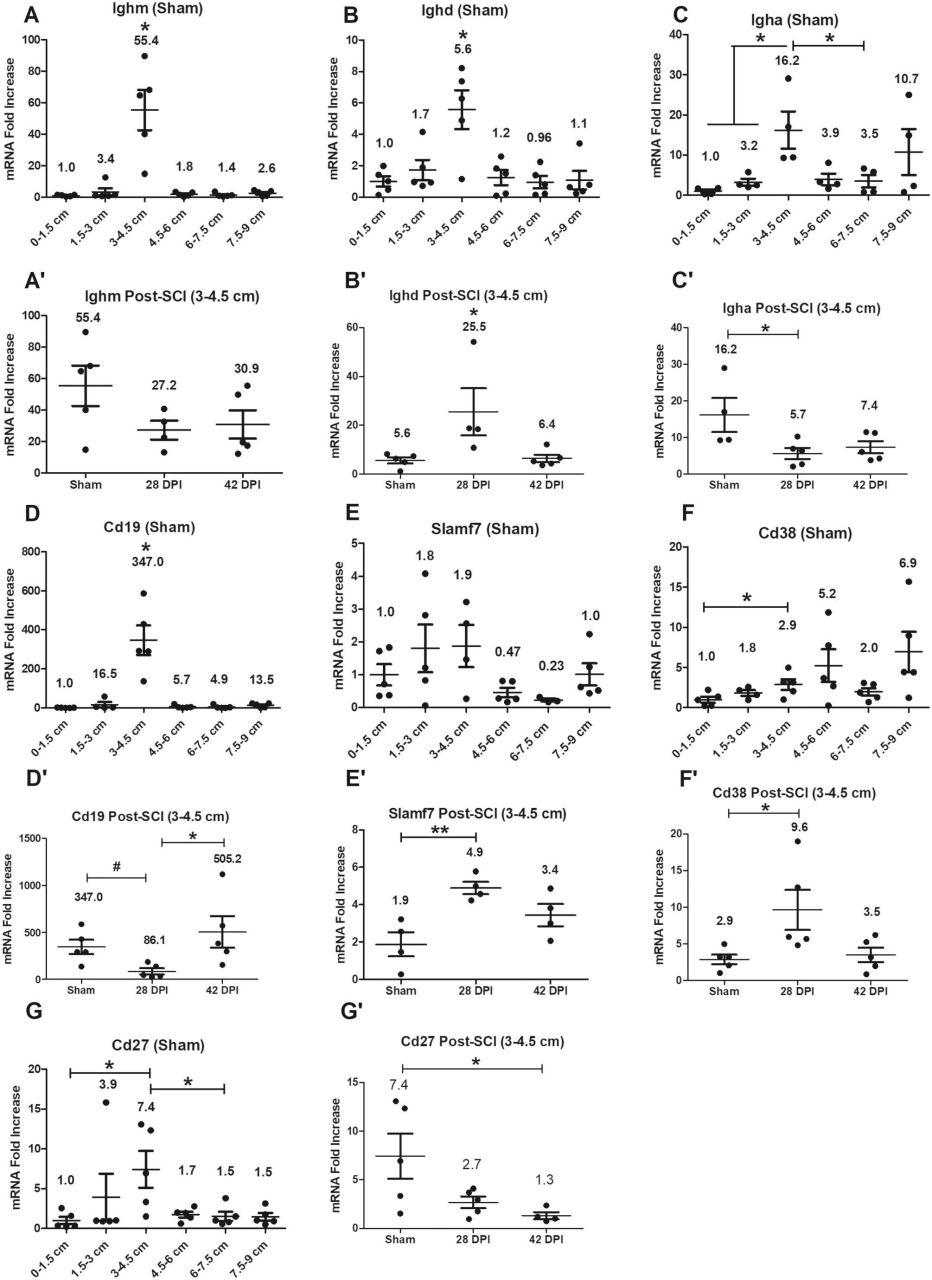
Gene expression levels of B cell-related genes. Gene expression levels of B cell-related genes were examined on six 1.5 cm long tissue segments of the anorectum for sham as well as 28 DPI and 42 DPI rats. After normalization to the 0–1.5 cm segment, the comparisons of gene expression levels between 3–4.5 cm segment and other 5 segments are analyzed by using paired *t* tests; and gene expression levels at the 3–4.5 cm segment between sham and injured groups are analyzed by using ANOVA with Tukey’s post hoc test correction (marked as *) and unpaired t tests (marked as #). Data are expressed as mean ± SEM (n = 6). Groups differ significantly (*or # *P* < 0.05, **P < 0.01).

In sham rats, RT-PCR revealed significantly higher gene expression levels of Ighm, Ighd, Igha, Cd19, Cd38 and Cd27 at the 3–4.5 cm anorectal segment from the dentate line, compared to levels at some or all the other segments (Fig. 2A–G). Due to the higher expression levels of B cell-related genes within the 3–4.5 cm anorectal segment of shams, these tissues were subsequently quantified in the SCI groups at two time points post-injury and compared relative to sham control levels. After SCI (Fig. 2B’–F’), compared to sham and/or 42 DPI, gene expression levels of Ighd and plasma markers Slamf7 and Cd38 in 28 DPI were significantly upregulated, whereas Igha and Cd19 were significantly downregulated. In contrast, there were no significant segment differences for Ighm (Fig. 2A’). However, the gene expression level of memory B cell marker Cd27 was significantly downregulated at 42 DPI relative to shams (Fig. 2G’). The data taken together indicate that the 3–4.5 cm segment of the rectum is a specific region where B cells/plasma cells accumulate, and their activities are impacted by SCI.

### Gene expression levels of other lymphoid cell markers such as CD4^+^ T cell-related genes

**Figure 3.**
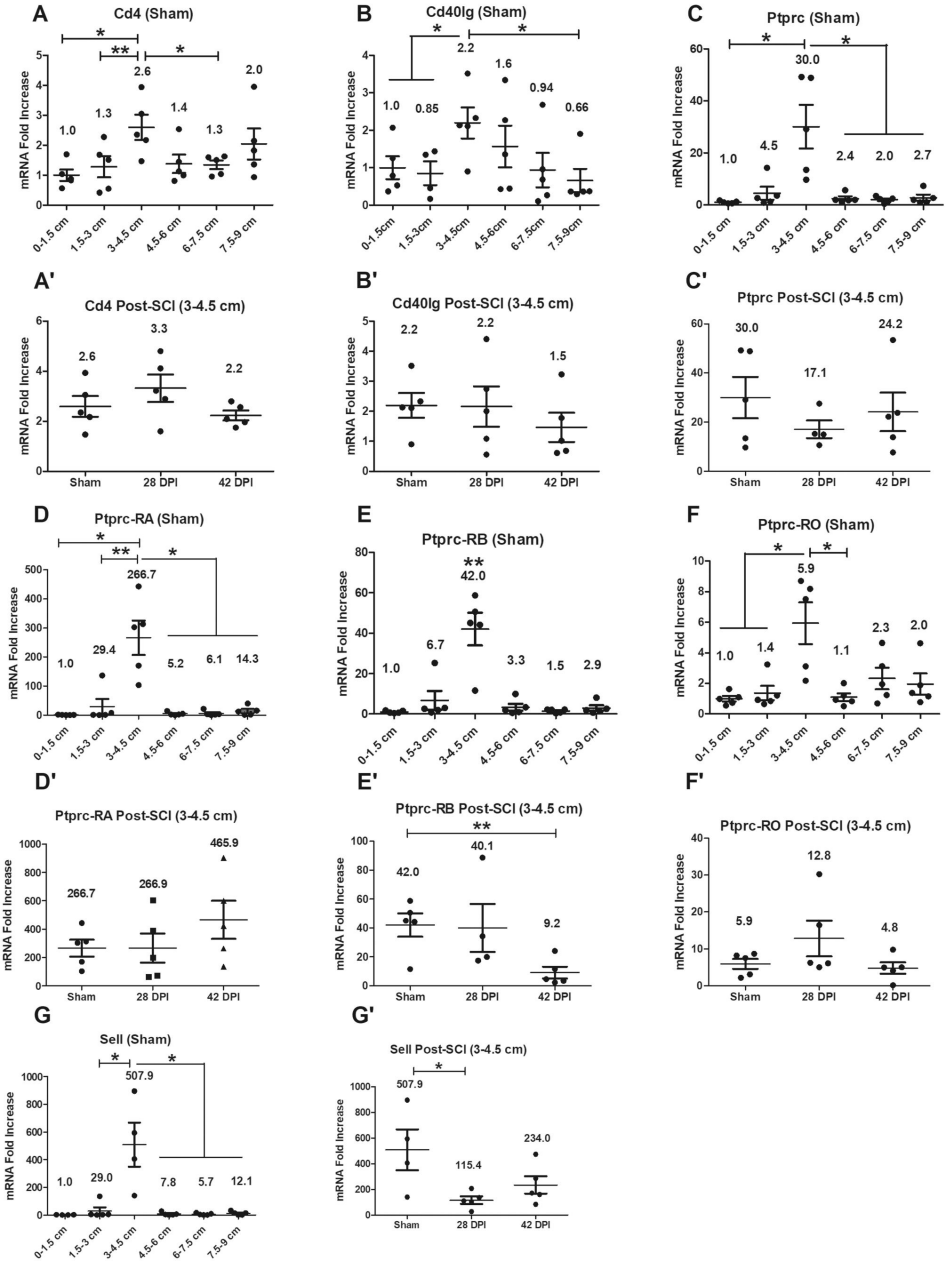
Gene expression levels of other lymphoid cell markers such as CD4^+^ T cell-related genes. Gene expression levels of other lymphoid cell markers were examined on six 1.5 cm long tissue segments of the anorectum for sham as well as 28 DPI and 42 DPI rats. After normalization to the 0–1.5 cm segment, the comparisons of gene expression levels between 3–4.5 cm segment and other 5 segments are analyzed by using paired *t* tests; and gene expression levels at the 3–4.5 cm segment between sham and injured groups are analyzed by using ANOVA with Tukey’s post hoc test correction (marked as *) and unpaired *t* tests (marked as #). Data are expressed as mean ± SEM (n = 6). Groups differ significantly (* *P* < 0.05, ** or ## *P* < 0.01).

Gene expression levels of CD4^+^ T cell and B cell-related genes were examined initially in all anorectal segments of sham animals. RT-PCR showed significantly higher gene expression levels for Cd4, Cd40lg, Ptprc (Cd45), Ptprc-RA (Cd45RA), Ptprc-RB (Cd45RB), Ptprc-RO (Cd45RO) and Sell (Cd62l) at the 3–4.5 cm segment of the six examined compared to levels at some or all other segments (Fig. 3A–G). After SCI, gene expression levels of Ptprc-RB and Sell were significantly downregulated at 42 DPI or 28 DPI respectively compared to sham. There were no significant changes for other targets (Fig. 3A’–G’).

### Gene expression levels of major histocompatibility complex (MHC) class II molecules and Ccr6

**Figure 4.**
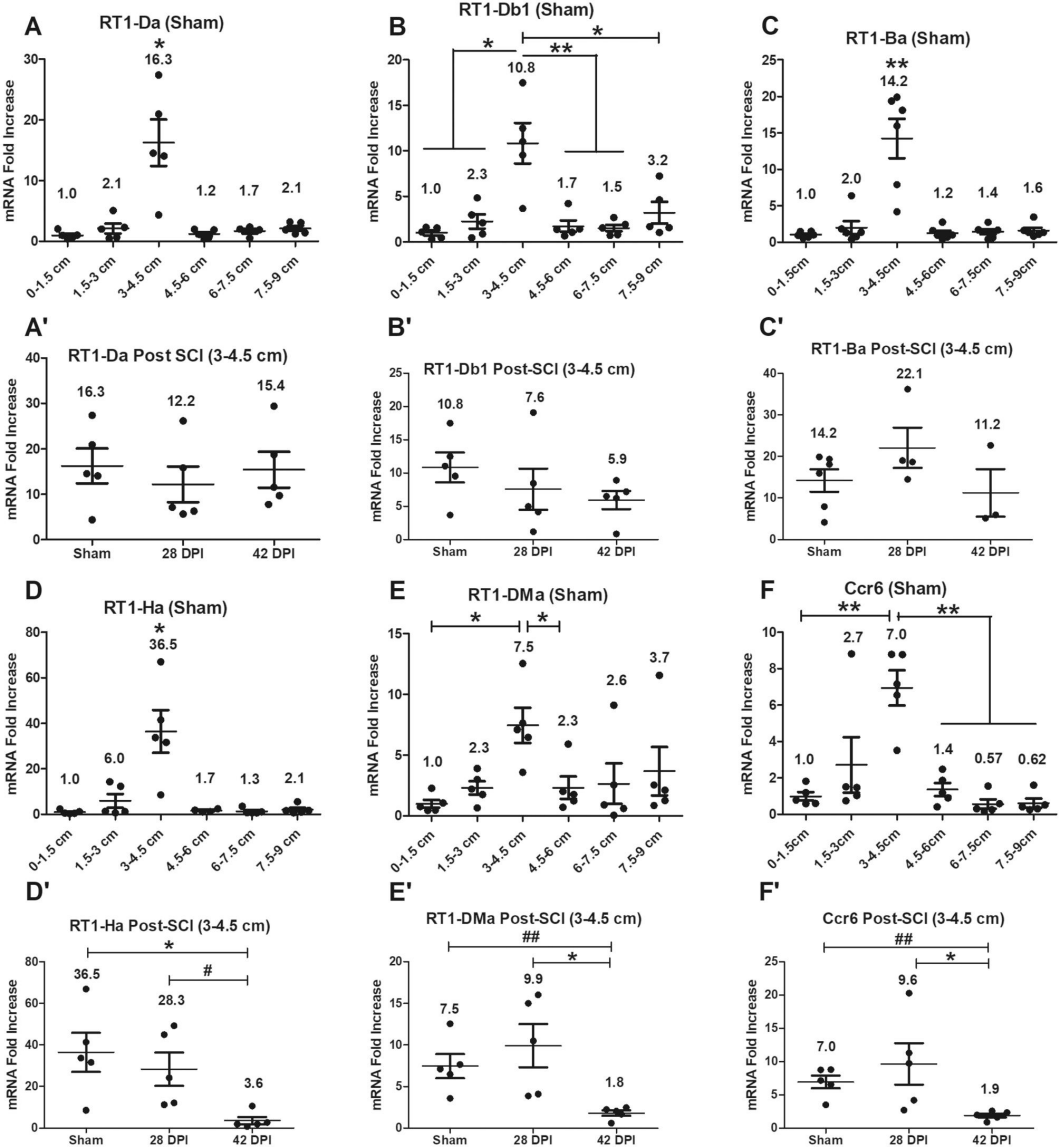
Gene expression levels of MHC class II molecules and Ccr6. Gene expression levels of MHC class II molecules and Ccr6 were examined on six 1.5 cm long tissue segments of the anorectum for sham as well as 28 DPI and 42 DPI rats. After normalization to the 0–1.5 cm segment, the comparisons of gene expression levels between 3–4.5 cm segment and other 5 segments are analyzed by using paired *t* tests; and gene expression levels at the 3–4.5 cm segment between sham and injured groups are analyzed by using ANOVA with Tukey’s post hoc test correction (marked as *) and unpaired t tests (marked as #). Data are expressed as mean ± SEM (n = 6). Groups differ significantly (*or # *P* < 0.05, ** or ## *P* < 0.01).

Antigen-presenting cells (APCs) use MHC class II molecules to bind to and present exogenous antigens, which are recognized by CD4^+^ T cells through binding between T cell receptor (TCR)-CD4 and antigen-MHC class II complex. Gene expression levels of MHC class II molecules and Ccr6 were examined initially in all anorectal segments of sham animals. RT-PCR showed significantly higher gene expression levels for all biomarkers in the 3–4.5 cm segment (Fig. 4A–F). After SCI, RT-PCR data indicate that gene expression levels of RT1-Ha, RT1-DMa and CCr6 were significantly downregulated at 42 DPI compared to sham and/or 28 DPI (Fig. 4A’–F’). There were no significant changes for RT1-Da, RT1-Db1 and RT1-Ba. The data indicate that the 3–4.5 cm segment of the rectum is also a specific region where APCs accumulate, and their activities are impacted/impaired by SCI.

### Gene expression levels of germinal center (GC)-related genes

**Figure 5.**
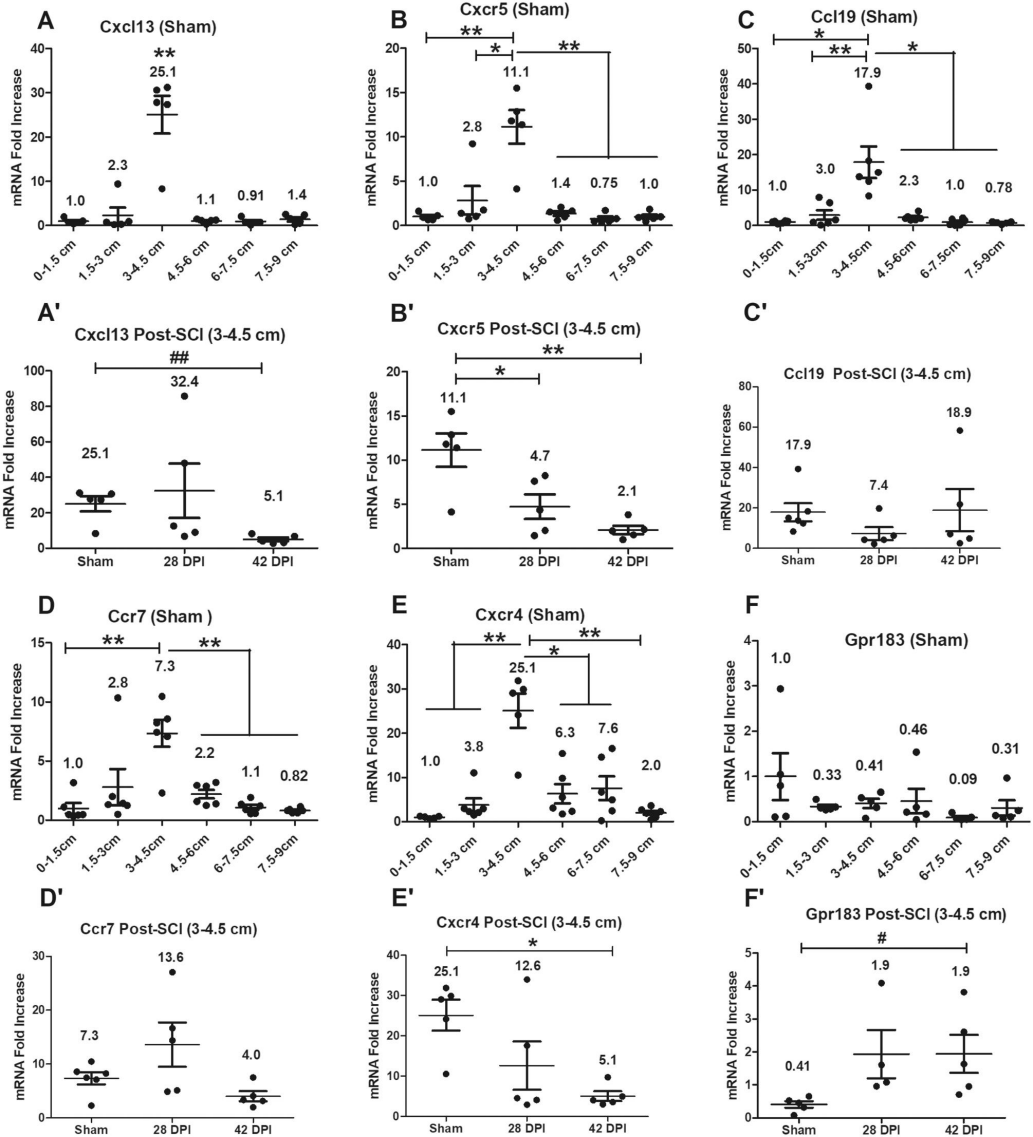
Gene expression levels of germinal center (GC)-related genes. Gene expression levels of germinal center (GC)-related genes were examined on six 1.5 cm long tissue segments of the anorectum for sham as well as 28 DPI and 42 DPI rats. After normalization to the 0–1.5 cm segment, the comparisons of gene expression levels between 3–4.5 cm segment and other 5 segments are analyzed by using paired *t* tests; and gene expression levels at the 3–4.5 cm segment between sham and injured groups are analyzed by using ANOVA with Tukey’s post hoc test correction (marked as *) and unpaired t tests (marked as #). Data are expressed as mean ± SEM (n = 6). Groups differ significantly (*or # *P* < 0.05, **or ## *P* < 0.01).

Gene expression levels of germinal center (GC)-related genes were examined initially in all anorectal segments of sham animals. RT-PCR revealed significantly higher gene expression levels for all targets except Gpr183 (Ebi2) at the segment 3–4.5 cm compared to some or all the other segments (Fig. 5A–F). Within the 3–4.5 cm anorectal segments after SCI relative to shams, gene expression levels were significantly downregulated at 42 DPI for Cxcl13 and Cxcr4, and at both 28 DPI and 42 DPI for Cxcr5, whereas the level of Gpr183 was significantly upregulated only in the 42 DPI group. In addition, there were no significant changes for Ccl19 and Ccr7 (Fig. 5A’–F’).

### Gene expression levels of proliferation and apoptosis-related genes and cytokine genes

**Figure 6.**
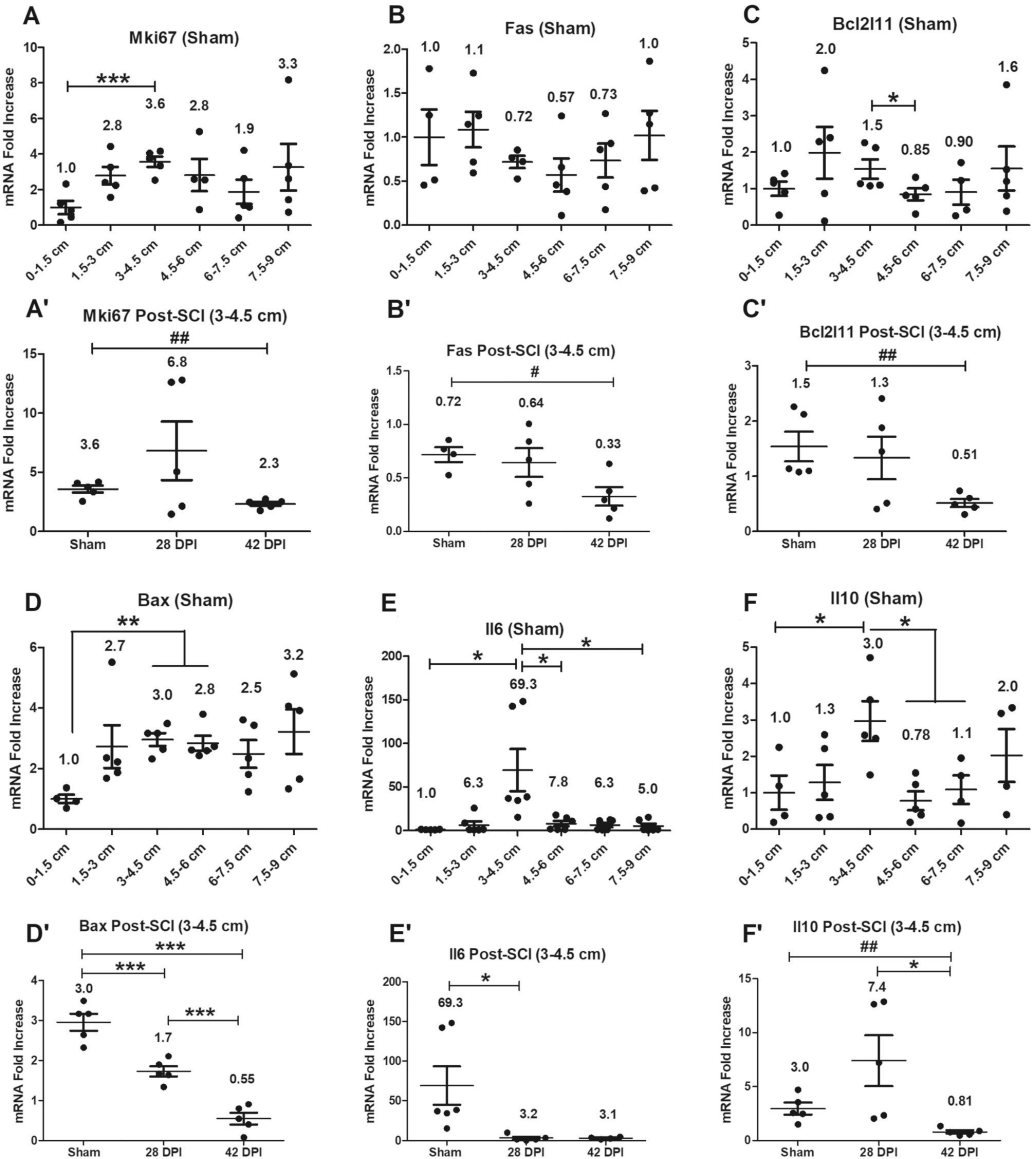
Gene expression levels of proliferation/apoptosis-related genes and cytokines. Gene expression levels of proliferation/apoptosis-related genes and cytokines were examined on six 1.5 cm long tissue segments of the anorectum for sham as well as 28 DPI and 42 DPI rats. After normalization to the 0–1.5 cm segment, the comparisons of gene expression levels between 3–4.5 cm segment and other 5 segments are analyzed by using paired t tests; and gene expression levels at the 3–4.5 cm segment between sham and injured groups are analyzed by using ANOVA with Tukey’s post-hoc test correction (marked as *) and unpaired *t* tests (marked as #). Data are expressed as mean ± SEM (n = 6). Groups differ significantly (*or # *P* < 0.05, **or ## *P* < 0.01, *** *P* < 0.001).

Expression levels of proliferation-related gene Mki67, apoptosis-related genes Fas (Cd95), Bcl2l11(Bim) and Bax as well as cytokines Il6 and Il10 were examined initially in all anorectal segments of sham animals. RT-PCR revealed significantly higher gene expression levels of all except Fas at the segment 3-4.5 cm from the dentate line, compared to some of the other segments (Fig. 6A–F). After SCI, gene expression levels of Mki67, Fas, Bcl2l11 and Il10 at the 3-4.5 cm segment were significantly downregulated at 42 DPI compared to shams and/or 28 DPI (Fig. 6A’–C’, F’); while gene expression level of Il6 was significantly downregulated at 28 DPI compared to shams (Fig. 6E’). In contrast, gene expression level of Bax was significantly downregulated at both 28 DPI and 42 DPI compared to shams and 28 DPI respectively (Fig. 6D’).

### Gene expression levels of inflammation-related genes

**Figure 7.**
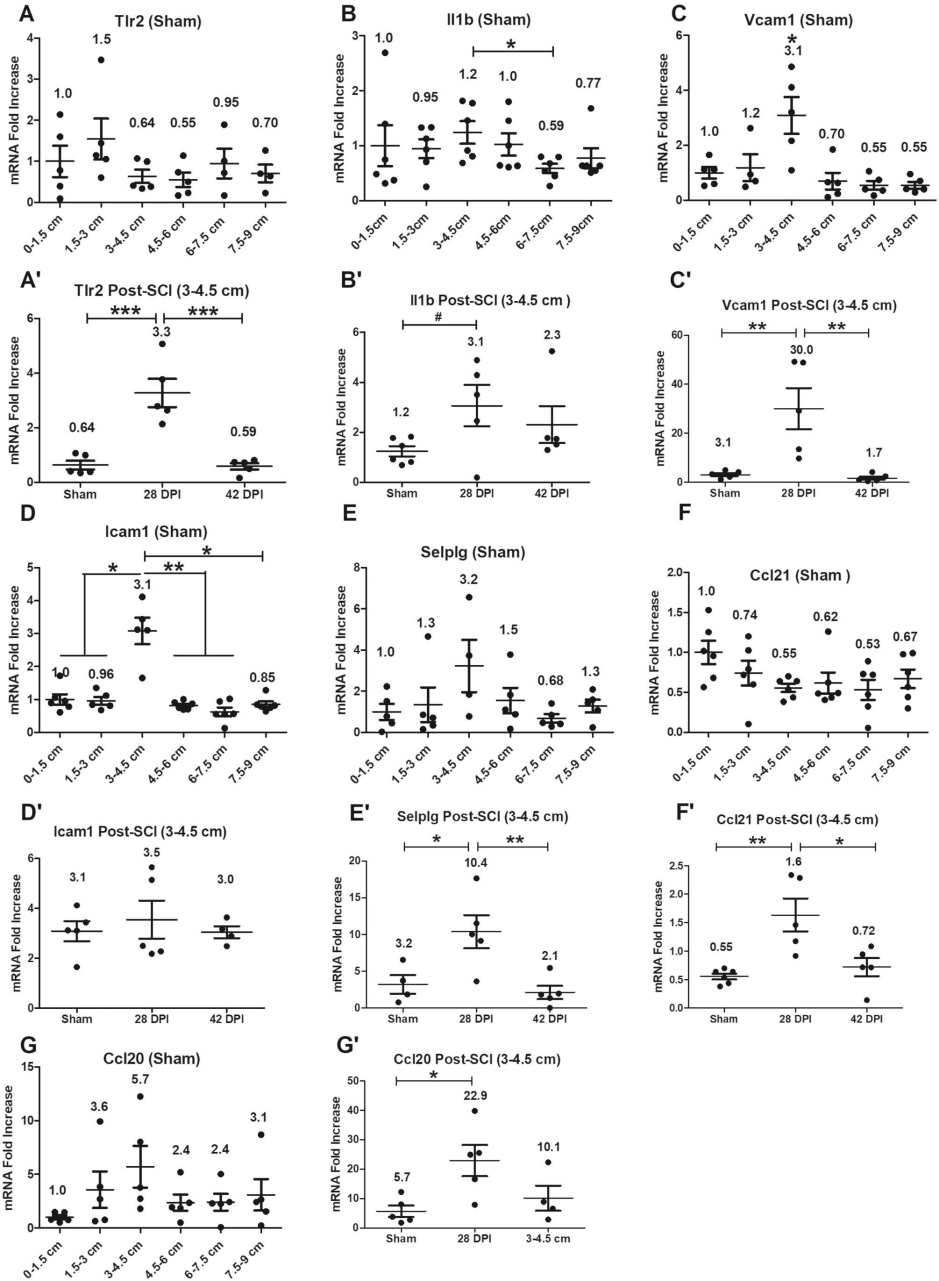
Gene expression levels of inflammation-related genes. Gene expression levels of inflammation-related genes were examined on six 1.5 cm long tissue segments of the anorectum for sham as well as 28 DPI and 42 DPI rats. After normalization to the 0–1.5 cm segment, the comparisons of gene expression levels between 3–4.5 cm segment and other 5 segments are analyzed by using paired t tests; and gene expression levels at the 3–4.5 cm segment between sham and injured groups are analyzed by using ANOVA with Tukey’s post-hoc test correction (marked as *) and unpaired t tests (marked as #). Data are expressed as mean ± SEM (n = 6). Groups differ significantly (*or # *P* < 0.05, **P < 0.01, *** *P* < 0.001).

Gene expression levels of inflammation-related genes Tlr2, Il1b, Vcam1, Icam1, Selplg (Selectin P ligand), Ccl21 and Ccl20 were examined initially in all anorectal segments of sham animals. RT-PCR showed significantly higher gene expression level of inflammation gatekeeper genes Il1b, Vcam1 and Icam1 at the 3–4.5 cm segment compared to some or all other segments (Fig. 7A–G). There were no significant changes for other targets. After SCI, gene expression levels of inflammation-related genes Tlr2, Il1b, Vcam1, Selplg, Ccl21 and Ccl20 were significantly upregulated at 28 DPI compared to shams and/or 42 DPI (Fig. 7A’–G’), with no significant changes for Icam1.

### Presence of rGALTs at the 3–4.5 cm within the rectum by histological staining

**Figure 8.**
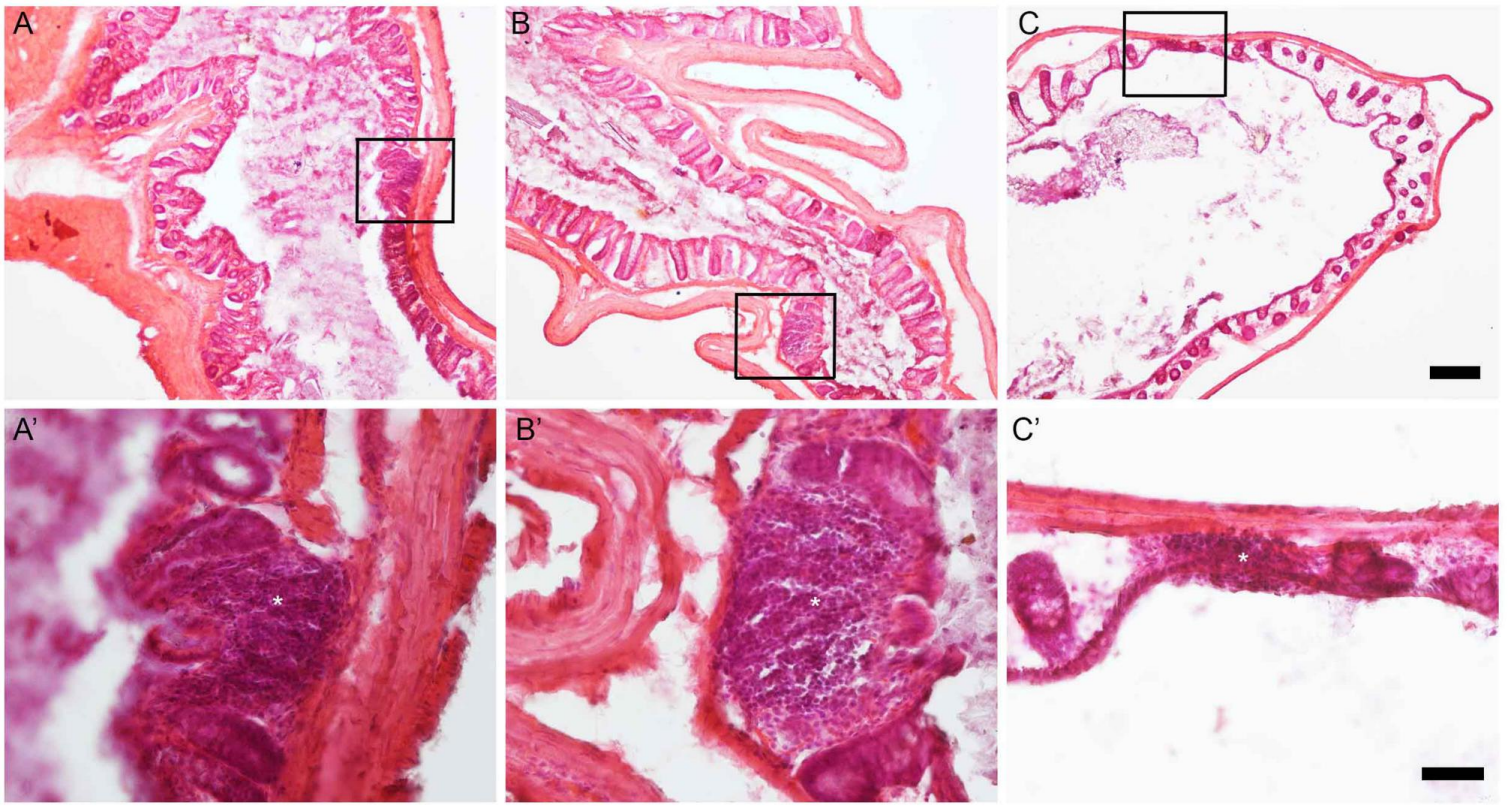
Hematoxylin and eosin staining (HE staining) of rGALT in the 3–4.5 cm segment of rectal tissue. Representative HE staining of transverse section of rectal tissue (3–4.5 cm) showed rGALT in sham rat (A, A’), 28 DPI (B, B’) and 42 DPI (C, C’) respectively. Aggregates of rGALT in the upper panel (box in A, B, C; 1.5 times of 4X) were shown in the lower panel in a higher magnification (A’, B’, C’; 1.5 times of 20X); asterisks mark centers of rGALT aggregates. Scale bars are 200 µm in the upper panel and 50 µm in the lower panel.

**Figure 9.**
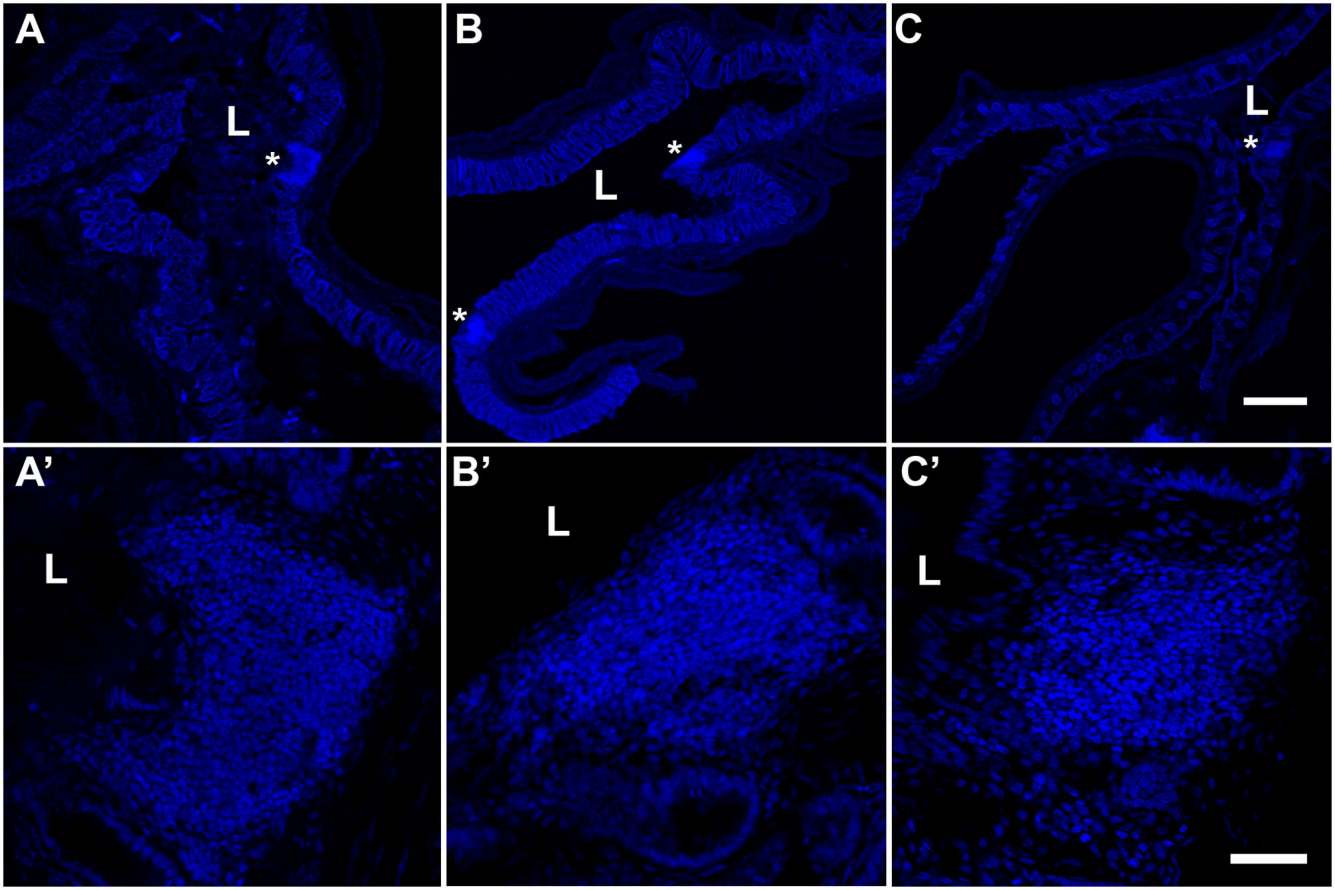
Nuclear staining of rGALT at the 3–4.5 cm segment of rectal tissue. Rectal tissues were stained with Hoechst 33342 to show nuclear staining of rGALT aggregates from the 3-4.5 cm segment in sham rats (A, A’), 28 DPI (B, B’) and 42 DPI (C, C’). rGALTs in the upper panel (A, B, C) (4X magnification) were shown at a higher magnification (20X) in the lower panel (A’, B’, C’); at 28 DPI, there were two aggregates of rGALT in B, the top rGALT in B was shown in B’. “L”s mark lumen side of the rectum; asterisks (*) mark the localization of rGALT in the upper panel. Scale bars are 200 µm in the upper panel and 50 µm in the lower panel.

Transverse sections of the 3–4.5 cm segment were stained with hematoxylin and eosin (HE) or with Hoechst 33342, respectively. HE staining and nuclear staining of rectal tissue (3-4.5 cm region) shows aggregates of rGALTs residing in the lamina propria of mucosa and/or submucosa in the rectal tissues from shams, 28 DPI and 42 DPI^44^^,^^47^^,^^60^ (Fig. 8 and Fig. 9). The morphological changes in microvillus surface/brush border were observed between sham and SCI groups (Fig. 8).

## Discussion

Data collected from spinally intact control group rats, when considered as a whole with respect to B cell and T cell-related markers, MHC class II molecules and GC-related genes, indicate that the presence of local immune tissue/organ rGALTs in the anorectum with localization primarily in the segment 3–4.5 cm (from the dentate line)^52^^,^^53^ (Fig. 1). Following T9 contusion, within the 3–4.5 cm tissue segment, upregulated gene expression levels of inflammation-related genes occurred at 28 DPI and downregulated GC organization and MHC class II molecules-related genes occurred at 42 DPI relative to surgical sham controls as identified by RT-PCR, suggesting that SCI modified/impaired the structural organization and function of rGALTs for the development of SCI-related gut dysbiosis, NBD and systemic diseases.^82^

### Sham data

Collectively, the sham data, when considered with respect to B cell and T cell markers, markers of APCs, markers of GC organizers, chemokines and cytokines, differentiation, proliferation, apoptosis and intercellular adhesion molecules as well as histological evidence, supports the existence of local immune tissue/organ rGALTs within the rectum.^25^^,^^29^^,^^31^^,^^34–37^ First, upregulated gene expression levels of mature B cell markers Ighm, Ighd,^83^^,^^84^ B cell-related marker Igha, common B cell marker Cd19,^85^ plasma cell marker Cd38,^86^ memory B cell marker Cd27^87–89^ and early ASC fate commitment marker Sell^90^ in non-injured rats (sham controls) indicate the presence and accumulation of B cells^91–93^ and generation of IgA^+^ ASCs and IgA^+^ memory B cells within the 3–4.5 cm region^63^^,^^94^^,^^95^ as B cells are important for organogenesis of the mucosal immune barriers.^96^ Second, PTPRC (CD45) is a tyrosine phosphatase expressed in alternatively spliced forms (isoform) on the surface of B and T cells; and Ptprc expression has been shown to be essential regulator for T cell^97^^,^^98^ and B cell development^99^^,^^100^ as well as T/B cell antigen receptor-mediated activation.^101–103^ Meanwhile, naïve CD4^+^ T cell could differentiate into helper T cells (TH) such as T follicular helper cells (TFH), which provide help to cognate B cells via their expression molecules such as Cd40lg^104^ and could differentiate into memory T cells expressing Ptprc-RO (Cd45RO).^104^^,^^105^ Accumulated lymphoid cells such as CD4^+^ T and B cells were further supported by upregulated gene expression levels of CD4^+^ T cell-related markers Cd4, mature B cell class-switch recombination (CSR) co-stimulatory molecule Cd40lg (Cd40l),^106–108^ Ptprc, naive T cell marker Ptprc-RA (Cd45RA),^101^ B cell differentiation marker Ptprc-RB (Cd45RB)^109^ as well as activated and memory T cell marker Ptprc-RO^110–112^ at the region of 3–4.5 cm tissue segment. Third, APCs were accumulated at the 3–4.5 cm tissue segment region, which was supported by upregulated gene expression levels of rat MHC class II molecules RT1-Da, RT1-Db1,^113^^,^^114^ RT1-Ba, RT1-Ha^115^, and RT1-DMa^116^^,^^117^ as MHC class II molecules are expressed only on the surface of APCs (macrophages, dendritic cells [DCs] and B cells). Fourth, GC contributes to cognate interaction between T and B lymphocytes to produce high-affinity antibodies against microbes and for the establishment of long-term immunological memory.^118–121^ GC-related genes Cxcl13/Cxcr5,^122–124^ Ccl19/Ccr7^125–127^ and Cxcr4 (128, 129) were upregulated at the 3–4.5 cm tissue segment.^130–132^ Fifth, other following biomarkers were also examined demonstrating that the 3–4.5 cm region was responsible for generation of IgA^+^ ASCs and IgA^+^ memory B cells^28^ for rectum homeostasis: (i) Mucosal lymphoid follicles in GALT are covered by a specialized follicle-associated epithelium (FAE) that contains microfold cells (M cells), which are at the antigen sampling zone sites and mediate uptake and transepithelial transport of luminal antigens.^133^ M-cell-mediated sampling of commensal bacteria is a required initial step for the efficient induction of intestinal sIgA.^35^ In shams, gene expression level of Ccr6 (43, 134, 135) was upregulated within rGALTs as expression of Ccr6 is critical for M cell formation inside PP,^136^^,^^137^ and via CCR6/CCL20 axis, activated B cells^138^/DCs^139^ migrate to the subepithelial dome (SED)^63^^,^^135^ of GALTs for acquiring gut-derived antigen uptake by M cells to trigger IgA immune response,^134^^,^^135^^,^^140^^,^^141^ and subsequently infiltrate pre-existing GCs for somatic hypermutation (SHM) and affinity-maturation.^129^^,^^142^^,^^143^ (ii) B cell selected for GC inclusion at the T-B border, having already gone through a round of division,^144^^,^^145^ are guided deep into the follicle dark zone (DZ) for further rounds of division/SHM and light zone (LZ) for affinity selection and apoptosis.^37^^,^^129^^,^^130^ In sham, gene expression levels of proliferation gene Mki67 (Ki67 nuclear antigen) and apoptosis-related gene Bax^146^^,^^147^ were upregulated within that region as, after antigen stimulation in SED, activated B cells are involved in initial proliferation of antigen-specific B cells, IgA class switch recombination (CSR), proliferation and selection of high-affinity B cell clones after entry into GCs,^106^^,^^130^^,^^145^ as well as apoptosis of autoreactive B clones during affinity selection.^129^^,^^130^^,^^143^ (iii) In sham, gene expression levels of Il6 and Il10 were upregulated within that region compared to other segments as IL6, as a growth factor for nonmalignant plasmablasts (PBs),^148^ supports stimulating differentiation of TFH cells^149^ and generation of long-lived plasma cells (LLPCs),^150^ and IL10 could induce differentiation of memory B cells to plasma cells in GC.^151^^,^^152^ (iv) Gene expression levels of intercellular adhesion molecule Vcam1^153^ and Icam1^154^ were upregulated at that region in sham as Vcam1 and Icam1 are expressed by non-hematopoietic lymphoid tissue stromal “organizer cells” (LTo) and is involved in the lymphoid organogenesis^102^^,^^155^^,^^156^ and leukocyte trafficking.^157^^,^^158^ Sixth, HE staining and nuclear staining in shams provided further evidence that organized rGALT aggregates existed in the mucosa and submucosa of rectal tissue segment 3-4.5 cm.^42^^,^^44^^,^^47^^,^^49^^,^^159^^,^^160^

**Figure 10.**
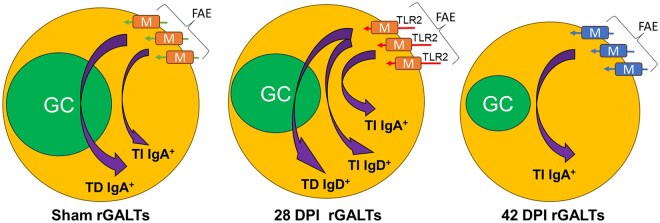
Proposed diagram for structure and function of rGALTs. In sham rats, rGALTs contains functional GC with both TD and TI IgA^+^ generation for producing IgA^+^ ASCs and IgA^+^ memory B cells (abbreviated as TD IgA^+^ and TI IgA^+^) following stimulation of gut-derived antigen uptake by M cells in FAE (green arrows). After SCI at 28 DPI, SCI induced upregulated TLR2 expression in M cells for an enhanced entry of microbiota to rGALTs (red arrows), which modified structure and function promoting EF responses in rGALTs. In additional to TI IgA^+^, rGALTs could contain both TD and TI IgD generation for producing IgD^+^ ASCs and IgD^+^ memory B cells (abbreviated as TD IgD^+^ and TI IgD^+^). At 42 DPI for resolution of inflammation, GC organization and differentiation of M cells were modified/impaired and EF responses were confirmed with and only TI IgA^+^ left (blue color). rGALTs, rectal gut-associated lymphoid tissues; GC, germinal center; ASC, antibody secreting plasma cell; TD IgA^+^, T cell-dependent IgA generation; TI IgA^+^, T cell-independent IgA generation; M cells, microfold cells; FAE, follicle-associated epithelium; TLR2, Toll-like receptor (TLR) 2; TI IgD^+^, T cell-dependent IgD generation; TD IgD^+^, T cell-dependent IgD generation; EF responses, extrafollicular B cell responses.

Collectively, the sham results indicate the existence of rGALTs in the rat rectum, which contain germinal center structure for TD IgA^+^ resembling other GALTs^31^^,^^47^^,^^49^ such as PP in the small intestine^25^ and CLPs in the colon.^42^^,^^53^ However, evidence also supports that the rGALTs could be a combination of rectal patches (GC resembling PP/CLPs) and SILTs (GC-absent)^43^^,^^44^^,^^60^^,^^160^ for generation of both TD IgA^+^ and TI IgA^+^ ASCs and IgA^+^ memory B cells^37^^,^^58^^,^^161^ (Fig. 10).

### SCI data at 28 DPI

At 28 DPI the data supports that SCI induced microbial infection and inflammation of rGALTs at 28 DPI, which promoted extrafollicular B cell responses (EF responses) in rGALTs and could elicit IgD CSR for generation of IgD^+^ ASCs and IgD^+^ memory B cells.

First, microbial infection and inflammation of rGALTs induced by SCI at 28 DPI are supported by multiple pieces of evidence including several upregulated inflammatory biomarkers (Fig. 7). (i) The rectum region contains the highest commensal microbiota and M cells within GALTs, which are associated with organized lymphoid tissue and could potentially provide vulnerable gateways for bacterial access across the robust epithelial barrier.^26^^,^^55^^,^^77^^,^^162^ Compared to sham and/or 42 DPI, upregulated gene expression levels of pathogen associated molecular pattern gene Tlr2^163^ and inflammation gatekeeper cytokine gene Il1b^164^ indicate microbial infection and inflammation of rGALTs as gut microbiota could induce TLR2 expression and activation^49^ at M cells resulting in microparticle (microbiota) entry to rGALTs in a dose-dependent manner.^165–167^ Furthermore, microbial infection and inflammation of rGALTs at 28 DPI are further supported by upregulated gene expression levels of Tlr2, Il1b, Selplg (Selectin P ligand) and Ccl20 within rGALTs on six 1.5 cm long tissue segments of the anorectum compared to some other segments (unpublished data). (ii) Inflammatory immune cells were recruited to rGALTs. Selplg is known to express on almost all leukocytes to provide the traffic signals (3 sequential steps: intercellular adhesion molecule, selectin and chemoattractant) for regulating leukocyte localization in the vasculature.^157^ Upregulated gene expression levels of Vcam1(inflammatory gatekeeper),^168^ Selplg^169^ and Ccl21^170^ indicate that lymphatic endothelial cells (LECs)^158^ of high endothelial venules (HEVs) and lymphatic vessels (LVs)^171^ within rGALTs could express Vcam1 and Ccl21 for recruiting inflammatory immune cells.^172^^,^^173^ Furthermore, upregulated gene expression level of Ccl21 could recruit naïve T cells, DCs^174^ and neutrophils^175^ through CCL21/CCR7 pathway. In addition to driving Ccr6^+^ B cells to the SED niche, upregulated CCL20/CCR6 axis could recruit other inflammatory immune cells such as NKT cells,^176^ DCs^177^, and monocytes.^178^

Second, microbial infection and inflammation of rGALTs impaired GC^82^ and induced B cell to migrate to extrafollicular (EF) regions with reducing TFH dependence for PB differentiation,^143^^,^^179^ which would generate short-lived plasma cells (SLPCs) and autoreactive B cells as the major function of the GC is to generate LLPCs and memory B cells for production of protective high-affinity antibodies.^30^^,^^180^ (i) Compared to sham, the upregulated gene expression level of Ccl20 at 28 DPI indicated that after an inflammatory stimulus,^177^ Ccl20 could be expressed by FAE enterocytes^181^^,^^182^ or stromal cells^183^^,^^184^ of SED of rGALTs and drove Ccr6^+^ B cells to the SED niche through the CCL20/CCR6 axis and reduced B cell GC infiltration.^43^^,^^134^^,^^135^ These changes could break immunological tolerance and induce EF responses^130^^,^^185^^,^^186^ as signals from cognate B cells play a critical role for further GC-TFH differentiation and the formation of GCs.^29^^,^^187^^,^^188^ (ii) TFH cells must express the chemokine receptor Cxcr5 for localization within B cell follicles to provide CD4^+^ T cell help^29^^,^^143^^,^^189^ and GC response was 2-fold reduced when T cells lacked CXCR5.^190^ Compared to sham, downregulated gene expression levels of Cxcr5 and Bax at 28 and 42 DPI, TFH differentiation factor Il6 (104, 149, 191) at 28 DPI indicated microbial infection and inflammation of rGALTs reduced antigen-driven-selection in the LZs of GC center^129^^,^^130^ and broke immunological tolerance for EF responses.^143^^,^^185^^,^^186^ Meanwhile, upregulated gene expression level of Selplg further supported induction of extrafollicular CD4^+^ T cells instead of TFH at 28 DPI as TFH cells are characterized by P-selectin glycoprotein ligand-1 (PSGL-1) downregulation,^192^ PSGL-1 distinguishes extrafollicular CD4^+^ T cells from follicular CD4^+^ T cells and PSGL-1^hi^ CD4^+^ cells reside only in the T cell zone and all GC CD4^+^ T cells are PSGL-1.^lo.187^

Third, microbial infection and inflammation of rGALTs could elicit IgD CSR for generation of IgD^+^ ASCs and IgD^+^ memory B cells within rGALTs for induction of systemic diseases in the whole body and gut dysbiosis in rectum. (i) microbial infection and inflammation of rGALTs could elicit IgD CSR and induce transition of PBs to IgD^+^ ASCs and IgD^+^ memory B cells at 28 DPI. Plasma cells could increase Cd38 expression and downregulated Cd19 expression;^85^^,^^193^^,^^194^ and lack of CD19 expression may be considered as a candidate marker for memory plasma cells maintaining long term memory during the transition of PBs to plasma cells.^193^^,^^195^ Thus, compared to sham, upregulated gene expression levels of Ighd, plasma cell markers Slamf7,^86^^,^^196^ Cd38 and downregulated gene expression levels of Igha and Cd19 (85) at 28 DPI indicate that microbial infection and inflammation of rGALTs could induce B cells differentiate and elicit IgD CSR for generation/form of IgD^+^ ASCs and IgD^+^ memory B cells^197^^,^^198^ instead of IgA CSR and IgA^+^ ASCs.^199^ This assumption was consistent with the notion that B cell CSR occurs mainly in EF region instead of GC^200^ and GALTS contains the molecular machinery to support TD and TI CSR^37^ as well as generate both TD IgD^+^ and TI IgD^+^ for production of IgD^+^ ASCs and IgD^+^ memory B cells (abbreviated as TI IgD^+^ and TD IgD^+^).^201^ This assumption was also supported by downregulated gene expression level of early ASC fate marker Sell (Cd62l)^90^ and the PB growth factor Il6 (148) at 28 DPI as well as upregulated memory plasma B marker Cd27 and memory B cell differentiation factor Il10 (151, 152) expression on six 1.5 cm long tissue segments of the anorectum at 28 DPI (our unpublished data) indicating that microbial infection and inflammation of rGALTs induced transition of PBs to IgD^+^ ASCs and IgD^+^ memory B cells at 28 DPI.^202^ (ii) Generation of IgD^+^ ASCs and IgD^+^ memory B cells at 28 DPI could induce systemic diseases in the whole-body system. Downregulated gene expression level of Cxcr5 at B cells^203^ and upregulated gene expression levels of adhesion molecules Vcam1 and Icam1^204^^,^^205^ could contribute to plasma cells such as IgD^+^ ASCs and IgD^+^ memory B cells of re-localization for shaping bone morrow (BM) pool and inducing systemic diseases.^62^^,^^63^^,^^65^^,^^194^^,^^206^ (iii) Generation of IgD^+^ ASCs and IgD^+^ memory B cells at 28 DPI could induce gut dysbiosis in rectum. Soluble dimeric IgA (dIgA) from ASCs is captured by polymeric immunoglobulin receptor (pIgR), which is on the basolateral surface of FAE and transcytosed to the apical surface of FAE and proteolytically cleaved to generate and release sIgA at the intestinal lumen.^207^ However, secreted IgD (sIgD) has limited access for coating/neutralization of gut microbiota due to its monomeric form and inability to be transported to gut lumen by pIgR,^198^^,^^208^ which would breakdown gut barrier and induce gut dysbiosis in rectum. Lack of coating gut microbiota would stimulate upregulated gene expression level of Tlr2 in M cells for enhanced microbiota entry into rGALTs, which supported generation of IgD^+^ ASCs and IgD^+^ memory B cells in a “SOS response” manner in rGALTs.^209^

Collectively, SCI induces microbial infection and inflammation of rGALTs, disrupts the cognate interaction between T and B cells favoring EF responses, and could promote IgD-BCR instead of IgA-BCR for generation of pathogenic IgD^+^ ASCs and IgD^+^ memory B cells.^143^^,^^161^ Thus, in addition to induction of systemic diseases, due to incapability to coat/neutralize gut microbiota, SCI would break out gut microbiota/barrier for development of SCI-related gut dysbiosis^208^ and contribute to changes of structure and function of rectal tissues including rGALTs^7^^,^^8^^,^^78^ (Fig. 10).

### SCI data at 42 DPI

At 42 DPI the data supports resolution of inflammation and modified structure and function of rGALTs for EF responses due to impaired gut-derived antigen acquirement and defective M cell differentiation as well as impaired GC organization in rGALTs.

First, M cell-mediated sampling of commensal bacteria was impaired at 42 DPI indicating impaired function/differentiation of M cells in rGALTs, which would compromise the function of rGALTs as acquiring gut-derived antigen uptake by M cells^96^^,^^210^ is required to trigger IgA immune responses. Antigens taken up by APCs are processed into peptide molecules, and then presented to CD4^+^ TH by MHC class II molecules on the cell surface; after antigen sensitization, MHC class II-antigen-restricted CD4^+^ TH can help generate TD IgA^+^ for specifically coating/neutralization of pathogen and microbes from gut. (i) compared to sham and/or 28 DPI, downregulated gene expression levels of MHC class II molecules RT1-Ha and RT1-DMa in 42 DPI indicate failure of antigen acquirement at rGALTs through APCs due to lack expression of relative MHC class II proteins on the surface of APCs as RT1-Ha is a cell surface receptor for antigens of foreign pathogens,^115^ and RT1-DM (DM), which is encoded by the genes RT1-DMa and RT1-DMb, can catalyze peptide acquisition of MHC class II molecules through the shaping of the presented peptidome with favoring the binding of high-affinity antigens.^116^^,^^117^^,^^211^ (ii) compared to sham, downregulated gene expression levels of Ccr6, B cell differentiation marker Ptprc-RB,^109^ memory plasma B marker Cd27 and its differentiation factor Il10 (143, 151, 152) at 42 DPI indicate impaired migration of B cells and impaired differentiation from B cells into ASCs and memory B cells at 42 DPI due to lack of expression of Ccr6 in B cells/DCs as via CCR6/CCL20 pathway migration of B cells/DCs to the SED of rGALTs is needed for acquiring gut-derived antigen,^134–137^^,^^140^^,^^141^ which support B cell EF responses at 42 DPI.

Second, at 42 DPI, GC organization was further modified/impaired. Compared to sham, downregulated gene expression levels of Cxcl13, Cxcr5 and Cxcr4 and upregulated follicle B cell segregation mediator Gpr183^212^^,^^213^ indicate impaired GC organization^128^^,^^129^ because: (i) follicular dendritic cells (FDC)^214^/TFH cells^179^ could produce Cxcl13 to recruit B cells into follicles;^215^ lack of Cxcr5 and Cxcl13 at 42 DPI could be due to lack of FDC or TFH cells. (ii) organization of GC is mediated by Cxcr4^216^ and Cxcr5;^142^^,^^191^ downregulated gene expression levels of Cxcr4 and Mki67 at 42 DPI could be due to reduced dividing centroblasts.^217^ (iii) proliferation and apoptosis-related genes Mki67 (Ki67 nuclear antigen),^144^^,^^145^ Fas (death receptor), Bcl2l11 (Bim) and Bax were downregulated at 42 DPI indicate impaired GC organization due to lack of negative and positive selection (apoptosis) at rGALTs^129^^,^^130^^,^^218^ as Fas^219^ could initiate extrinsic cell death pathway of autoreactive B cells and pro-apoptotic Bcl2l11^220^^,^^221^ could limit the survival of mature B cells in a cell-autonomous manner by engaging apoptotic activator Bax.^129^^,^^130^^,^^146^^,^^147^^,^^218^

Third, modified/impaired rGALTs at 42 DPI were further supported by no significant changes for genes Cd38, Cd27, Cd40lg, Ptprc-RB, Ptprc-RO, RT1-Ba, RT1-Ha, Ccl19, Bax, Il10 and Vcam1 at rGALTs on six 1.5 cm long tissue segments of the anorectum compared to other segments. Although SCI modified structure and function of rGALTs resulting in lack of GC-related TD IgA^+^, TI IgA^+^ could exist at 42 DPI because on six 1.5 cm long tissue segments of the anorectum compared to other segments, upregulated gene expression of lymphoid cell-related markers such as Ighm, Ighd, Igha, Cd19, Prprc and Prprc-RA, MHC class II molecules RT1-Da and RT1-Db as well as genes Sell, Cxcl13, Cxcr4, Mki67 and Il6 enriched at rGALTs indicating B cells were recruited for proliferation and TI IgA^+^ generation (our unpublished data).

Collectively, SCI impairs acquirement of gut-derived antigen and GC structure organization/function in rGALTs and induces compromised B cell differentiation for EF responses. GC-related TD IgA^+^ disappeared, whereas TI IgA^+^ could exist in rGALT at 42 DPI indicating that rGALTs generated autoreactive B cells and SLPCs with low-affinity sIgA at 42 DPI^32^^,^^34^^,^^143^^,^^161^ due to lack of positive and negative affinity selections, which support induction of gut dysbiosis in rectum and induction of systemic diseases in the whole-body system.

In summary, upregulated gene expression levels of immune biomarkers along the rat anorectum reveal the existence of rGALTs within the segment of 3–4.5 cm from the rectal dentate line. The current data further suggest that SCI could have an impact on B cell ability to mount T dependent antibody responses after injury, which was not suggested in the study of systemic immunity.^222^ After SCI, changes of biomarker expressions within rGALTs at the 28 and 42 DPI indicate modified/impaired structure and function of rGALTs due to microbial infection and inflammation,^26^^,^^42^^,^^47^^,^^162^ which are involved in the development of NBD and are consistent with our previous functional manometry data such as elevated mean baseline pressure, increased mean baseline contraction frequency and increased mean baseline area under the curve values after contusion at about that region (the difference of distances likely comes from point of measurement, ie in vivo study from the anal verge; while current study, from the rectal dentate line)^13^ (Fig. 10). In addition to the generation of pathogenic IgD^+^ ASCs and/or IgD^+^ memory B cells at 28 DPI,^223^^,^^224^ microbial infection and inflammation of rGALTs promoted EF responses instead of the initiation of the antigen-specific immune response due to modified/impaired structure and function of rGALTs at 42 DPI and/or 28 DPI. Although GALTs such as ILF in the colon often have small or absent germinal centers^54^ for generation of valuable TI IgA^+^ ASCs for broadly targeting non-invasive commensals,^32^^,^^58^^,^^225^^,^^226^ TD IgA^+^ ASCs within rGALTs can provide cognate T cell help for the production of high-affinity antibodies against microbes and for the establishment of long-term immunological memory in rectum, which play a critical role for maintaining the diversity and stability of commensal microbiota as well as coating penetrant commensals and invasive enteric pathogens and toxins.^30^^,^^33^^,^^34^^,^^49^^,^^208^^,^^227^ Thus, generation of pathogenic IgD-BCR, autoreactive B cells and SLPCs due to EF responses within rGALTs would break down the gut barrier and induce the local SCI-related gut dysbiosis as GC-related IgA^+^ ASCs and memory B cells produced by rGALTs serve as the first line barrier/defense for rectal homeostasis and could perform coating/neutralization of pathogen and microbes.^25^^,^^29–38^ Consequently, after SCI, the bidirectional interaction between impaired ENS and dysfunction of rGALTs likely contributes to the development of gut dysbiosis, structure and function changes of rectal tissues,^7^ and NBD^72^^,^^77^^,^^78^ (Fig. 8). Finally, both gut dysbiosis-induced translocation of gut pathological microorganisms^16^^,^^72^^,^^228^ and rGALTs’ shaping the whole-body B cell repertoire^62–65^ would further contribute to development of systemic diseases.

Study limitations were the number of post-SCI timepoints (2), 1 sex (male), and a single level and extent of injury. Future study will further explore early causal events such as the disorders of enteric nervous system after traumatic SCI in the development of NBD as well as the process of microbial infection and inflammation for modifying structure and function of rGALTs since the enteric nervous system of the gastrointestinal tract interacts with the local immune system bidirectionally.^8^^,^^78^ After we have identified the underlying mechanisms in the development of NBD, therapies could be developed to prevent second injury since gut dysbosis/NBD are disease modifying factors for traumatic SCI and other CNS diseases.

## Conclusion

There is a local immune tissue/organ rGALTs localized within the rectum at the 3–4.5 cm segment in rats. rGALTs, as an integral element, are responsible for generation of IgA^+^ ASCs and memory B cells in rectum for rectal gut homeostasis, first line barrier and defense, as well as for shaping the B cell repertoire of the whole body. However, rGALTs can be vulnerable gateways for bacterial access across the robust epithelial barrier leading to multiple systemic diseases, including autoimmune diseases. SCI induces microbial infection and inflammation of rectal tissues containing rGALTs, resulting in impairment of structure and function of rGALTs for generation of autoreactive B cells and SLPCs due to EF responses instead of the initiation of GC-related antigen-specific immune response for generation of LLPCs and memory B cells, which consequently breaks down homeostasis of rGALTs and rectum microbiota/barrier for the development of SCI-related gut dysbiosis, NBD, and systemic diseases.

## Institutional review board statement

The animal study protocol was approved by the Institutional Animal Care and Use Committee of University of Louisville.

## Data Availability

To request data for this study, contact Dr Charles Hubscher at chhubs01@louisville.edu.
